# Sublinear transport in Kagome metals from the interplay of Dirac cones and Van Hove singularities

**DOI:** 10.1038/s41467-025-65685-0

**Published:** 2025-12-05

**Authors:** Nikolai Peshcherenko, Ning Mao, Claudia Felser, Yang Zhang

**Affiliations:** 1 Max Planck Institute for Chemical Physics of Solids, Dresden, Germany; 2 Department of Physics and Astronomy, University of Tennessee, Knoxville, Tennessee USA; 3 Min H. Kao Department of Electrical Engineering and Computer Science, University of Tennessee, Knoxville, Tennessee USA

**Keywords:** Electronic properties and materials, Electronic structure

## Abstract

Recent experiments have revealed that many kagome metals exhibit sublinear temperature dependence in resistivity up to room temperature. Here, we develop a minimal semiclassical two-pocket model—comprising a Dirac cone and a Van Hove singularity—and show that, within an extended Fermi liquid scattering framework, internode electron-electron interactions naturally lead to sublinear scaling in both electrical and thermal transport at low temperatures. At higher temperatures, distinct scattering channels for charge and heat currents lead to a violation of the Wiedemann-Franz law. Our work provides a simple and broadly applicable framework for understanding anomalous transport in kagome metals, capturing non-Fermi-liquid behavior without requiring fine-tuning or exotic interactions.

## Abstract (web summary)

Many kagome metals exhibit a sublinear temperature dependence in resistivity up to room temperature. Here, the authors propose a semiclassical two-pocket theory consisting of a Dirac cone and Van Hove singularity to explain this behavior.

## Introduction

In itinerant metals, electrons/holes are the dominant charge carriers, and the Fermi liquid framework typically applies. Consequently, a clean system is expected to exhibit a resistivity scaling with *T*^2^ at intermediate temperatures, reflecting electron-electron interactions and signaling Fermi liquid behavior. The deviations from this well-known result are studied both experimentally and theoretically as the signatures of strongly interacting systems, including Luttinger liquid systems^1^, heavy fermion metals^2^, underdoped cuprates^3^, graphene at charge neutrality point^4,5^, etc. These deviations, in terms of resistivity scaling, could be both superlinear (meaning resistivity scaling with temperature *T* as *ρ*(*T*) ∝ *T*^*n*^, *n* > 1, *n* ≠ 2) and linear. The so-called strange metal state featuring *T* linear scaling was previously observed in refs. ^6–10^ and superlinear temperature behavior was predicted for materials in the vicinity of quantum phase transition^11,12^. Although these systems have quite different properties, the non-Fermi liquid behaviors are all linked with emerging collective excitations due to strong interactions.

Recently, Kagome lattices have emerged as pivotal platforms for exploring strongly correlated phenomenon^13,14^, topological magnetism^15–21^, and unconventional superconductivity^22^. Experiment on Kagome metal Ni_3_In^23^ has demonstrated a quite unexpected and well-pronounced sublinear in *T* resistivity (*ρ*(*T*) ∝ *T*^*n*^, *n* < 1) at relatively high temperatures *T* ≳ 100 K, where strong quantum fluctuations or collective excitations are unlikely to appear. Similar sublinear behavior, albeit less pronounced, has been observed in three-dimensional Kagome compounds like ScV_6_Sn_6_, CsV_3_Sb_5_, RbV_3_Sb_5_, KV_3_Sb_5_^24–30^ and Yb_0.5_Co_3_Ge_3_^31,32^, MgCo_6_Ge_6_^33^ around room temperature (see also Table 1). Therefore, the most plausible explanation for this type of behavior should rather be semiclassical. However, existing semiclassical scattering contributions from electron-phonon coupling predict *ρ*(*T*) ∝ *T*^*n*^, *n* ≥ 1^34,35^, and impurity scattering is known to give *T*-independent contribution.

**Table 1:** Temperature scaling exponent *γ* (from experimental data) and Van Hove singularity position with respect to the Fermi level (taken from DFT calculations) for certain materials from the kagome family

Material	Exponent *γ*, *ρ*(*T*) ∝ *T*^*γ*^	Temperature range, K	VHS position, meV	ARPES VHS signature	Effective mass, *m*_*e*_
Ni_3_In	0.3^23^	100–300	10^23^, Fig. 4	^23^	–
ScV_6_Sn_6_	0.6^24^	91–400	80^24^	^58^	0.5^59^
CsV_3_Sb_5_	0.6^24,25^	94–300	30^60^	^61,62^	0.03^63^
RbV_3_Sb_5_	0.6^24,28^	102–300	70^60^	^42,62^	0.1^28^
KV_3_Sb_5_	0.6^24,26^	78–300	60^60^	^64^	0.1^65^
Yb_0.5_Co_3_Ge_3_	0.6^31,32^	100–300	20^66^	–	–
MgCo_6_Ge_6_	0.8^33^	100–300	50^66^	–	–
Ni_3_Sn	1^23^	50–300	100^23^	^23^	–

The temperature range exhibiting nonlinear behavior is listed, with the upper temperature limit corresponding simply to the room temperature. The lower temperature limit for V-based materials corresponds to the charge density wave transition temperature that would bring^24^ the Fermi level closer to the VHS. For the VHS being sufficiently close to the Fermi level, *γ* < 1 due to enhanced electron-electron scattering. For a remote VHS, *γ* = 1 due to electron-phonon scattering^35^. Experimental evidence for the Van Hove singularity and for Dirac pocket (in the form of a small effective mass obtained from Shubnikov-de Haas measurement) is listed.

In this work, we introduce a two-pocket model consisting of Dirac cone and Van Hove singularity, which allows a sublinear in temperature scaling of resistivity as observed in the family of Kagome materials. Our model study focuses exclusively on single electron excitations, which are semiclassically described using the Boltzmann equation. The crucial ingredient is the internode electron-electron interaction, which is enhanced due to the divergent density of states from VHS at Fermi level. We demonstrate that even though this internode scattering conserves momentum, its presence alongside momentum-relaxing processes (like impurity scattering) generates a leading *T*-sublinear contribution to resistivity, with a different sublinear exponent also emerging for thermal conductivity. Furthermore, we show that this model predicts a breakdown of the Wiedemann-Franz law and a nontrivial temperature dependence of the Lorentz number. At temperatures higher than Dirac node chemical potential, both electrons and holes are thermally activated near the Dirac node. And the intranode scattering in Dirac systems is able to relax electric but not thermal current. Thus, thermal current is relaxed only with internode scattering. In our setup, the dominant electric and thermal carriers are essentially the same, but they are relaxed by intranode and internode scattering, respectively.

## Results

### Two-pocket model

The abundant exotic states in Kagome materials are deeply linked to their remarkable band structures. These band structures consist of a Dirac node, a saddle point Van Hove singularity (VHS) and a flat band^36–38^, which was proved by both density functional theory calculations^26,39–41^ as well as by experimental evidence such as ARPES^25,42^ and scanning tunneling microscope spectroscopy. For the relevant material examples, also see Table 1.

**Fig. 1:**
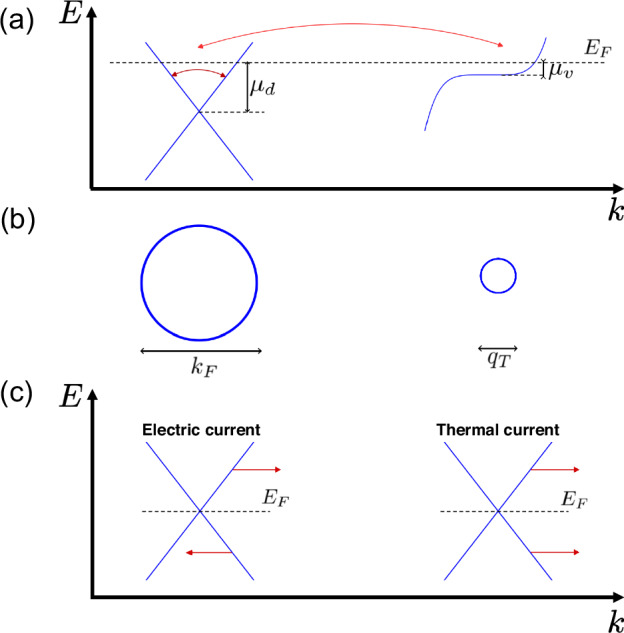
Schematics for a kagome metal band structure. **a** Two-pocket model of a Kagome metal. Node *d* is a Dirac pocket with a relatively large Fermi surface. Fast Dirac electrons relax due to both intranode (shown as dark red arrow) and internode (bright red) electron-electron scattering. Node *v* has weak dispersion *E*_*v*_ ~ *Ak*^*α*^, *α* > 2 and *μ*_*d*_, *μ*_*v*_ are local chemical potential. **b** Thermally activated Fermi pockets for *μ*_*v*_ ≪ *T* ≪ *μ*_*d*_. For *T* ≫ *μ*_*v*_ the effective size of Fermi surface *q*_*T*_ (which is the size of the thermally activated electrons cloud in the momentum space close to the VHS) at node *v* is *T*-dependent: $A{q}_{T}^{\alpha }\lesssim T$, or, equivalently, ${q}_{T}\lesssim {\left(T/A\right)}^{1/\alpha }$. Due to the weak dispersion of VHS electrons, fast Dirac electrons scattering process is almost energy-conserving. This internode process is dominant due to high *ν*_*v*_ and allows for sublinear *T* dependence. **c** For *T* ≫ *μ*_*d*_, intranode scattering is able to relax only charge but not thermal current. Here, an electron-hole pair taken at the same momentum provides opposite in sign contributions to charge transport, but the same sign for heat transport. Thus, contribution to heat transport is proportional to total momentum and could not be relaxed by intranode scattering^52^.

We start with an explicit introduction of a two-pocket model (see Fig. 1), as flat band is generally away from Fermi level. The fast pocket is basically a Dirac pocket with an isotropic Dirac spectrum

1 $${E}_{d}({{{\bf{k}}}})={v}_{F}| {{{\bf{k}}}}|$$

and the slow pocket has saddle point type of momentum dispersion in *k*_*z*_ = 0 plane:

2 $${E}_{v}({{{\bf{k}}}})=A\left(| {k}_{x}{| }^{\alpha }-| {k}_{y}{| }^{\alpha }\right).$$

We denote two pockets as *d* (Dirac) and *v* (VHS), correspondingly. It is important to note that in our model, the *v* pocket refers only to thermally activated electrons closest to the VHS, with the exact size *q*_*T*_ of the relevant electronic cloud to be defined below. Although the full Fermi pocket near the VHS can be much larger, only electrons in the immediate vicinity of the VHS are relevant for the scattering channel considered in this work.

Density of electronic states from node *vν*_*v*_(*E*) demonstrates VHS behavior:

3 $${\nu }_{v}(E)=	 \sum \limits_{{{{\bf{k}}}}}\delta \left(E-{E}_{v}({{{\bf{k}}}})\right)\propto \frac{1}{\alpha A}{\left(\frac{A}{| E| }\right)}^{\beta },\\ \beta=	 1-\frac{2}{\alpha }.$$

for *α* > 2. Since having a singular density of states is crucial for our analysis, in what follows, we consider exclusively *α* > 2. This saddle point VHS is located near *M* momentum in Kagome metals such as Ni_3_In and ScV_6_Sn_6_ (see discussion in “Experimental data comparison” section and in Supplementary Information, section II). Since *v* electrons near the VHS are slow (have relatively small Fermi velocities), all transport currents are mainly carried by fast Dirac electrons *d*.

However, *v* pocket electrons still influence transport properties. This is due to their high density of states, which enhances the scattering rate of internode electron-electron interactions. These interactions in the experimentally relevant limit *T* ≫ *μ*_*v*_^23,24^ could manifest themselves in sublinear temperature scaling of kinetic coefficients, with *μ*_*v*_ being defined at Fig. 1a. Namely, for interpocket electron-electron scattering, the electrons closest to the Van Hove singularity provide the leading contribution (due to singular density of states) to the scattering rate. In the limit *T* ≫ *μ*_*v*_, the size of the thermally activated electrons cloud near VHS in momentum space is *q*_*T*_ ∝ *T*^1/*α*^ (see description for Fig. 1). At the same time, *E*_*v*_(**q**) demonstrates relatively weak dispersion in the vicinity of the Van Hove singularity. Thus, the scattering of Dirac electrons at *v* pocket electrons proceeds with negligible energy transfer. This is why *v* electrons could be treated as a momentum-relaxing reservoir with its size *q*_*T*_ dependent on *T*. We show below that the observed sublinear *ρ*(*T*) behavior^23–28^ stems from this *T*-dependent effective *v* pocket size *q*_*T*_.

We begin with a detailed study of the relatively large Dirac Fermi surface limit for *T* ≪ *μ*_*d*_, so that a standard low-*T* quasiparticle picture holds. Nevertheless, we also give consideration to *T* ≫ *μ*_*d*_ case as it is experimentally accessible and, as we will show below, reveals additional observable physical phenomena.

### Boltzmann equation description

Fast current-carrying Dirac electrons distribution function *f*_*d*_ obeys semiclassical Boltzmann equation:

4 $${\partial }_{t}{f}_{d}+{{{\bf{v}}}}\cdot {\partial }_{{{{\bf{r}}}}}{f}_{d}+e{{{\bf{E}}}}\cdot {\partial }_{{{{\bf{p}}}}}{f}_{d}=	 {I}_{{{{\rm{intra}}}}}+{I}_{{{{\rm{inter}}}}}+\\ 	+{I}_{{{{\rm{imp}}}}}+{I}_{{{{\rm{nc}}}}},$$

where *I*_intra_, *I*_inter_ stand for intra- and internode electron-electron scattering, *I*_imp_ is for impurity scattering, and *I*_nc_ describes other types of momentum-relaxing contributions (e.g., scattering at phonons or Umklapp processes for electron-electron interactions).

Below Debye temperature in Kagome metals^23–28^ e-e interaction appears to play a major role in transport phenomena. As we show in what follows, this is due to the sublinear scaling of scattering rate with temperature being contributed to exclusively by e-e scattering. Other types of contributions, such as electron-phonon or impurity scattering, provide only superlinear *T* dependence^34^ for electron-phonon exchange and *T* independent contribution for impurity scattering. Since in metallic systems, electron-electron interaction is screened, we model it in the form of contact interaction with amplitude *g*:

5 $${H}_{{{{\rm{int}}}}}({{{\bf{r}}}},{{{{\bf{r}}}}}^{{\prime} })=g\delta \left({{{\bf{r}}}}-{{{{\bf{r}}}}}^{{\prime} }\right),$$

with an exception for *T* ≫ *μ*_*d*_ case (see below). This assumption is justified for typical momentum transfers that are small compared to the inverse screening length. Umklapp processes for electron-electron scattering are very rare due to the very large momentum transfer required and are therefore omitted.

**Fig. 2:**
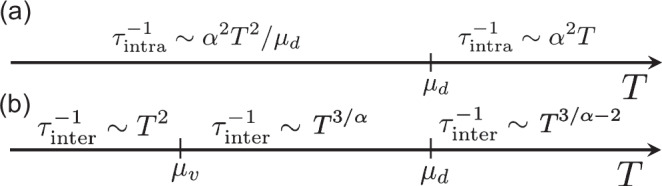
Intra- and internode scattering rates for fast current-carrying Dirac electrons. Electron-electron scattering rate as a function of temperature for **a** Dirac intranode scattering and **b** internode scattering processes. For *T* ≫ *μ*_*v*_, the effective size of *v* pocket is *T*-dependent, which gives rise to sublinear *T* dependence. For *T* ≪ *μ*_*d*_ internode scattering is dominant due to high density of states in *v* pocket. However, for *T* ≫ *μ*_*d*_ intranode scattering is stronger since intranode interactions are not screened. Coupling constant *α* = *e*^2^/*ℏv*_*F*_ is assumed to be small.

Electron-electron scattering processes could be basically of two types: intranode (happening between electrons from the same node) or internode (between different nodes), for an outline see Fig. 2. We tackle these two processes separately.

### Intranode scattering

We first note that intranode processes within *v* pocket are not relevant to transport properties, since the corresponding electronic states possess low group velocity and thus provide only a small direct contribution to transport currents. Therefore, we focus on Dirac electrons scattering. The corresponding scattering rate was previously evaluated in refs. ^43–45^. Result for electron-electron relaxation time depends on the relation between Dirac fermions chemical potential *μ*_*d*_ (see Fig. 1a) and temperature *T*. For relaxation rate ${\tau }_{{{{\rm{intra}}}}}^{-1}$ we have:

6 $${\tau }_{{{{\rm{intra}}}}}^{-1}\propto {\left(\frac{{e}^{2}}{\hslash {v}_{F}}\right)}^{2}\left\{\begin{array}{ll}\frac{{T}^{2}}{{\mu }_{d}},\quad &T\ll {\mu }_{d}\\ T,\quad &T\gg {\mu }_{d}\end{array}\right.,$$

so that a crossover between Fermi (∝*T*^2^) and high temperature behavior happens at *T* ~ *μ*_*d*_.

### Internode scattering

Internode electron-electron scattering appears to be a crucial ingredient for sublinear *T* behavior of transport coefficients. As we show below, this behavior is due to the weakly dispersing VHS band. Namely, due to the weak dispersion of these states, internode scattering of fast current-carrying Dirac electrons proceeds with negligible energy transfer. Thus, the expression for the internode transport scattering rate takes the following simple form:

7 $$\frac{1}{{\tau }_{{{{\rm{e}}}}}-{{{\rm{e}}}}} \sim {n}_{v}{g}^{2}{\sum}_{{{{\bf{q}}}}}\delta \left(E({{{\bf{p}}}})-E({{{\bf{p}}}}+{{{\bf{q}}}})\right)(1-\cos {\theta }_{{{{\bf{p+q}}}},{{{\bf{p}}}}}),$$

where *θ*_**p+q,p**_ describes Dirac electron’s scattering angle between its initial **p** and final **p + q** momenta, *n*_*v*_ is the concentration of VHS electrons. The phase volume of scattered electrons in Eq. (7) is, however, limited by the magnitude of transferred momentum **q**. The upper limit for **q** could be, in turn, estimated by the size of momentum-relaxing reservoir of VHS states *q*_*T*_ ≲ (*T*/*A*)^1/*α*^ (see Fig. 1b). It is important to note that even for an anisotropic Van Hove singularity, the estimate for the transmitted momentum *q*_*T*_ ∝ *T*^1/*α*^ would hold true, allowing for sublinear resistivity scaling discussed below. Assuming small momentum transfer *q*_*T*_ ≪ *p*_*F*_ (which is true for *T* ≪ *μ*_*d*_), the scattering angle *θ*_**p+q,p**_ could be estimated as *θ*_**p+q,p**_ ~ *q*/*p* ≪ 1, so that for the scattering rate, one arrives at

8 $$\frac{1}{{\tau }_{{{{\rm{e}}}}}-{{{\rm{e}}}}} \sim \frac{{g}^{2}{n}_{v}}{{\mu }_{d}^{2}/{v}_{F}}{\left(\frac{T}{A}\right)}^{3/\alpha }.$$

Eq. (8) is the main finding of the present work (for the more detailed evaluation, please see Supplementary Information, section I). Namely, this VHS-mediated electron-electron scattering leads to sublinear scaling of transport coefficients.

Let us emphasize that the obtained result is valid only in the limit of Van Hove singularities being sufficiently close to the Fermi level (*T* ≫ *μ*_*v*_). At this point, it is worth noting that it is challenging to verify whether this condition is truly met. From an experimental perspective, a highly accurate (precision not worse than 10 meV) ARPES measurement is needed. Theoretical DFT predictions may be inaccurate due to ambiguous doping or strain that would shift the Fermi level in a way specific to the experimental setup in question. Nonetheless, in what follows, we demonstrate that the applicability of our model treatment to real systems could be tested in various thermoelectric measurements.

### Momentum-relaxing processes and sublinear *T* behavior

One should, however, be aware that momentum-conserving electron-electron interactions alone would lead to non-decaying current; thus, a minimal transport estimation of Kagome metals should include momentum-relaxing processes. As experimental estimates for ScV_6_Sn_6_^24^ show, impurity scattering exhibits the shortest scattering time among momentum-relaxing processes. Electron-phonon interactions prove to be negligible^24^, since sublinear *T* behavior is observed below the Debye temperature. Thus, the minimal relaxation term should include internode electron-electron scattering and impurity relaxation.

Within this minimal model of scattering, one could calculate both electrical *σ* and thermal *κ* conductivities for *T* ≪ *μ*_*d*_ by solving the Boltzmann equation (4):

9 $$\begin{array}{rcl}\sigma &=&{e}^{2}{\nu }_{d}({\mu }_{d})D,\quad \kappa=\frac{{\pi }^{2}}{3}{\nu }_{d}({\mu }_{d})DT,\hfill \\ D&=&\frac{1}{2}{v}_{F}^{2}{\tau }_{{{{\rm{eff}}}}}({\mu }_{d},T),\quad {\tau }_{{{{\rm{eff}}}}}={\left({\tau }_{{{{\rm{imp}}}}}^{-1}+{\tau }_{{{{\rm{e}}}}}^{-1}-{{{\rm{e}}}}\right)}^{-1}.\end{array}$$

**Fig. 3:**
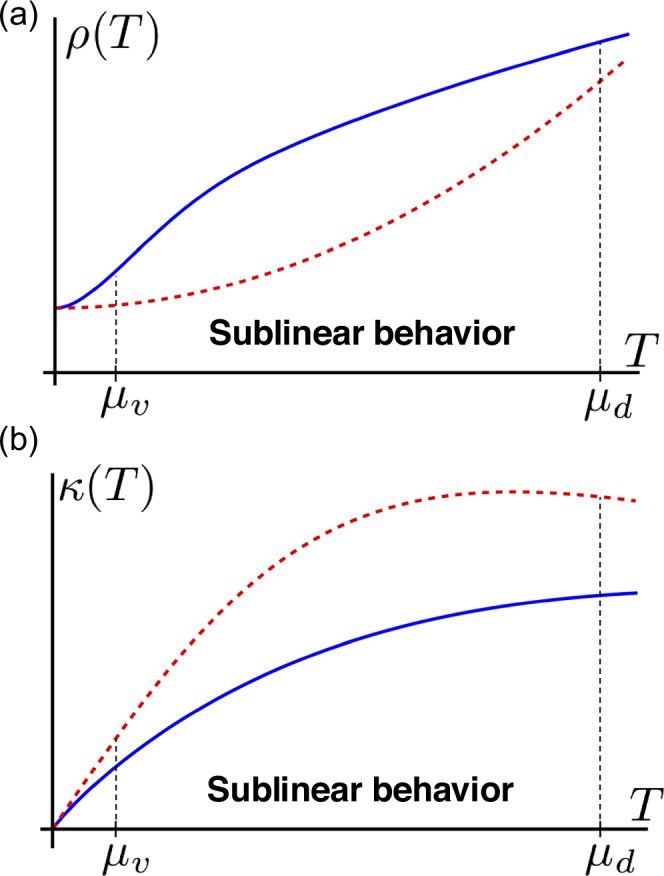
Predicted transport coefficients sublinear scaling with temperature. **a** *T*-dependent resistivity *ρ*(*T*) for the two-pocket model of a Kagome metal (solid blue line) and a standard Fermi liquid behavior (dashed red line). Kagome metals' resistivity demonstrates sublinear temperature behavior *ρ*(*T*) ∝ *T*^3/*α*^ in a range *μ*_*v*_ ≪ *T* ≪ *μ*_*d*_ due to the dominant internode electron-electron scattering rate given by Eq. (8). For large *T* ≫ *μ*_*d*_, intranode interaction becomes the strongest due to lack of screening and thus *ρ*(*T*) ∝ *T*. **b** *T*-dependent thermal conductivity *κ*(*T*). Since for *T* ≪ *μ*_*d*_ Wiedemann-Franz law holds, *κ*(*T*) also has a sublinear behavior as *κ*(*T*) ∝ *T*^1−3/*α*^ in the range *μ*_*v*_ ≪ *T* ≪ *μ*_*d*_. For *T* ≫ *μ*_*d*_, however, Wiedemann-Franz law breaks down due to the separation of electric and thermal current relaxation channels (see discussion below).

We plot the resistivity and thermal conductivity in Fig. 3. Despite demonstrating non-Fermi liquid-like behavior of sublinear *T*-dependence, Eq. (9) suggests that the Wiedemann-Franz law still holds. This is because both charge and heat currents decay due to the same process, namely, internode electron-electron scattering. However, as we discuss later, for high temperatures *T* ≫ *μ*_*d*_ it is not so, and the Wiedemann-Franz law breaks down.

It is worth mentioning that our predictions could be tested for both thermal conductivity and thermopower measurement. The thermopower, due to Mott relation, is expected under certain conditions (band structure and impurity scattering specific) to demonstrate the same sublinear scaling with thermal conductivity  ∝ *T*^1−3/*α*^. According to recent experimental studies, the predicted sublinear behavior was realized in CsV_3_Sb_5_ compound^46^. For ScV_6_Sn_6_^47^, however, other contributions were dominant, providing for linear behavior.

### Comparison with experimental data

As mentioned in the “Introduction,” the well-pronounced sublinear in temperature resistivity was observed^23^ in Ni_3_In compound for *T* ≳ 100 K. In the subsequent sections, we illustrate how our two-pocket semiclassical model offers a plausible explanation for this phenomenon.

**Fig. 4:**
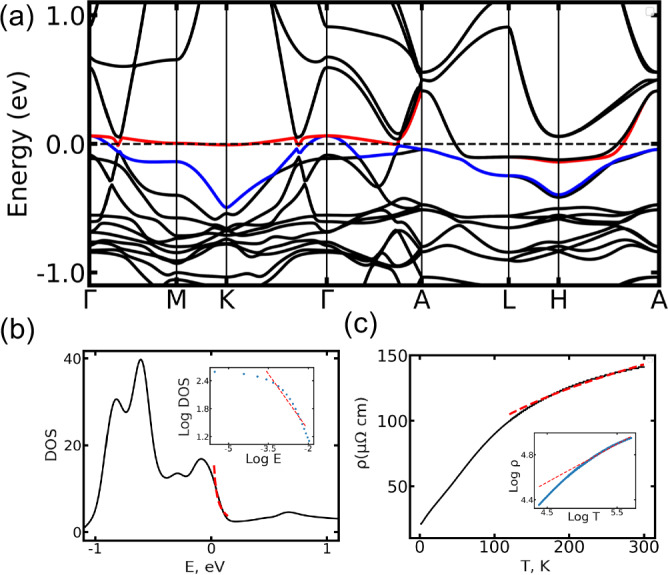
First-principles and experimental resistivity scaling with temperature for Ni_3_In. **a** DFT electronic structure of Ni_3_In. Fermi level is shown by a dashed line. Red and blue colors represent the two bands closest to the Fermi level. **b** DFT total density of states shows a peak near Fermi level. Dashed red line stands for the fitting as ${(E-{E}_{{{{\rm{VHS}}}}})}^{-\beta }$, *β* = 0.8 (please see inset for log log plot), *E*_VHS_ = 0. **c** Ni_3_In experimental resistivity *ρ*(*T*)^23^ versus temperature is shown. Sublinear fitting of Ni_3_In resistivity *ρ*(*T*) ∝ *T*^*γ*^ gives ${\gamma }_{\exp }=0.34$ (red dashed line and inset log-log plot) in accord with our theoretical prediction.

Band structure calculation for Ni_3_In (see Fig. 4a) identifies the fast Dirac pocket near *Γ* point. Further, the DFT density of states (see Fig. 4b) demonstrates a peak around Fermi energy. Density of states power law fitting DOS$(E)\propto {(E-{E}_{{{{\rm{VHS}}}}})}^{-\beta }$ leads to *β* = 0.8, a power-law divergent higher order Van Hove singularity^48–50^. According to Eqs. (3) and (8), this predicts resistivity exponent ${\gamma }_{{{{\rm{th}}}}}=\frac{3}{2}(1-\beta )=0.3$ (*ρ*(*T*) ∝ *T*^*γ*^). This agrees well with the experimental data fitting result ${\gamma }_{\exp }=0.34$. We note the suggested mechanism does not describe the resistivity curve in Fig. 4c below  ~100 K. As discussed in ref. ^23^, below 100 K, the resistivity is dominated by strongly non-Fermi liquid correlations. However, for higher temperatures, this correlated state is claimed to be destroyed, falling into the semiclassical regime.

We further perform DFT calculations for other Kagome compounds such as ScV_6_Sn_6_, CsV_3_Sb_5_, RbV_3_Sb_5_, KV_3_Sb_5_. In Supplementary Information, section II, we prove with the help of **k ⋅ p** model fitting that experimentally observed^24^ universal sublinear resistivity behavior agrees well with our theory prediction.

### Wiedemann-Franz law breakdown

**Fig. 5:**
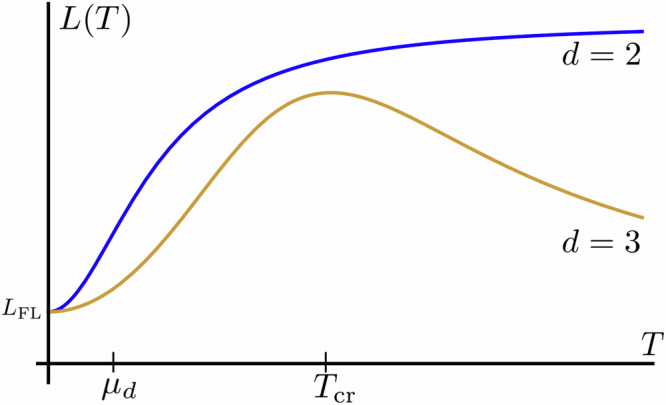
Lorentz number *L*(*T*) behavior with temperature *T.* For *T* ≪ *μ*_*d*_, Wiedemann-Franz law holds because both charge and heat current relax due to almost elastic internode e-e scattering. For *T* ≫ *μ*_*d*_, it breaks down since although internode e-e interactions are the strongest, they do not provide thermal current relaxation for *T* ≫ *μ*_*d*_. For *μ*_*d*_ < *T* < *T*_cr_, thermal current relaxation is provided by internode e-e scattering, which gives nontrivial power-law behavior *L*(*T*) ∝ *T*^*δ*^, *δ* = 3 − 3/*α*. For higher temperatures *T* > *T*_cr_, the increase of *L*(*T*) would be cut due to energy-dependent for *T* ≫ *μ*_*d*_ impurity scattering (the higher temperature behavior also depends on the dimension). ${L}_{{{{\rm{FL}}}}}=\frac{{\pi }^{2}}{3}{\left(\frac{{k}_{B}}{e}\right)}^{2}$ is Fermi liquid value of Lorentz number. *T*_cr_ is a crossover temperature between *τ*_inter_ and *τ*_imp_ dominated heat current relaxation.

Another interesting aspect of the two-pocket model is Wiedemann-Franz law violation at high temperatures case (*T* ≥ *μ*_*d*_). The electron-hole symmetry is approximately restored within this limit for Dirac node, which essentially implies the absence of screening for intranode interactions (screening length^51^${l}_{{{{\rm{scr}}}}}^{2}\propto 1/\nu ({\mu }_{d})\to \infty$). This, in turn, makes the quantitative theoretical description of transport phenomena cumbersome. Hence, the aim of this section is not to give accurate derivations but rather reasonable estimates for the behavior of Lorentz number *L*(*T*) with temperature. For *L*(*T*), one could write

10 $$\frac{L(T)}{{L}_{{{{\rm{FL}}}}}} \sim \frac{{\tau }_{\kappa }}{{\tau }_{{{{\rm{intra}}}}}},$$

where ${\tau }_{\kappa }=\min \{{\tau }_{{{{\rm{inter}}}}},\,{\tau }_{{{{\rm{imp}}}}}\}$ describes the thermal current relaxation time. According to ref. ^52^, intranode electron-electron scattering is unable to relax thermal current (see also Fig. 1c). It is therefore done by internode and impurity scattering. Charge current relaxation time is still given by the shortest time scale (which is now *τ*_intra_ due to the absence of screening under approximate electron-hole symmetry). The separation of thermal and charge currents relaxation channels is the ultimate reason for Wiedemann-Franz law breakdown in this limit. Our mechanism is thus in a sense different from previously discussed Wiedemann-Franz law violation in cuprates due to spin-charge separation^53–55^. For the schematic plot of *L*(*T*) behavior, please see Fig. 5.

## Discussion

In this work, we have proposed a semiclassical theory to explain sublinear scaling of charge and heat transport coefficients, based on a two-pocket model (Dirac node + Van Hove singularity) of Kagome metals. When Fermi level is sufficiently close to VHS, the dominant scattering source of current-carrying Dirac electrons is internode electron-electron scattering. We have demonstrated that electronic states near high-order Van Hove singularity effectively form a momentum-relaxing reservoir for fast Dirac electrons, allowing only for a vanishing energy exchange. The reservoir size is though *T*-dependent: ${q}_{{{{\rm{VHS}}}}}\lesssim {\left(T/A\right)}^{1/\alpha }$. Combined with a relatively large Dirac pocket (*q*_*D*_ ≫ *q*_VHS_) or generic fast electron pocket, this allows for a sublinear *T*-dependence of transport coefficients, *ρ*(*T*) ∝ *T*^3/*α*^, *κ* ∝ *T*^1−3/*α*^. Our finding for resistivity scaling directly explains the recent transport experiments in Kagome metal^23,24^. Nevertheless, despite the sublinear behavior at relatively low temperatures, Wiedemann-Franz law still holds since both charge and heat currents relax due to the same internode scattering process. While for relatively large temperatures, the thermal and electric current relaxation channels split due to the approximate electron-hole symmetry of Dirac node^52^, leading to Wiedemann-Franz law violation.

## Methods

We performed density functional theory (DFT) calculations using the Full-Potential Local-Orbital (FPLO) code, with the generalized gradient approximation (GGA) in the Perdew-Burke-Ernzerhof (PBE) formulation^56,57^. For the numerical evaluation of the density of states, we employed a dense 250  × 250  × 250 k-point mesh in the Brillouin zone.

## Supplementary information

Supplementary Information

Transparent Peer Review file

## Data Availability

The data that support the findings of this study are available from the corresponding author upon request.

## References

[1] Wakeham, N. et al. Gross violation of the Wiedemann–Franz law in a quasi-one-dimensional conductor. *Nat. Commun.* **2**, 396 (2011). 21772267 10.1038/ncomms1406 PMC3144592

[2] Tanatar, M. A., Paglione, J., Petrovic, C. & Taillefer, L. Anisotropic violation of the Wiedemann-Franz law at a quantum critical point. *Science* **316**, 1320 (2007). 17540899 10.1126/science.1140762

[3] Hill, R., Proust, C., Taillefer, L., Fournier, P. & Greene, R. Breakdown of Fermi-liquid theory in a copper-oxide superconductor. *Nature* **414**, 711 (2001). 11742390 10.1038/414711a

[4] Crossno, J. et al. Observation of the Dirac fluid and the breakdown of the Wiedemann-Franz law in graphene. *Science* **351**, 1058 (2016). 26912362 10.1126/science.aad0343

[5] Müller, M., Fritz, L. & Sachdev, S. Quantum-critical relativistic magnetotransport in graphene. *Phys. Rev. B* **78**, 115406 (2008).

[6] Fournier, P. et al. Insulator-metal crossover near optimal doping in Pr_2-x_Ce_x_CuO_4_: anomalous normal-state low temperature resistivity. *Phys. Rev. Lett.* **81**, 4720 (1998).

[7] Sarkar, T. et al. Fermi surface reconstruction and anomalous low-temperature resistivity in electron-doped La_2-x_Ce_x_CuO_4_. *Phys. Rev. B* **96**, 155449 (2017).

[8] Martin, S., Fiory, A. T., Fleming, R., Schneemeyer, L. & Waszczak, J. V. Normal-state transport properties of Bi_2+x_Sr_2-y_CuO_6+__*δ*_ crystals. *Phys. Rev. B* **41**, 846 (1990). 10.1103/physrevb.41.846 9992839

[9] Cooper, R. A. et al. Anomalous criticality in the electrical resistivity of La_2–x_Sr_x_CuO_4_. *Science* **323**, 603 (2009). 19074310 10.1126/science.1165015

[10] Daou, R. et al. Linear temperature dependence of resistivity and change in the Fermi surface at the pseudogap critical point of a high-*T*c superconductor. *Nat. Phys.* **5**, 31 (2009).

[11] Maslov, D. L., Yudson, V. I. & Chubukov, A. V. Resistivity of a non–Galilean–invariant Fermi liquid near Pomeranchuk quantum criticality. *Phys. Rev. Lett.* **106**, 106403 (2011). 21469816 10.1103/PhysRevLett.106.106403

[12] Smith, R. et al. Marginal breakdown of the Fermi-liquid state on the border of metallic ferromagnetism. *Nature* **455**, 1220 (2008).

[13] Wang, W.-S., Li, Z.-Z., Xiang, Y.-Y. & Wang, Q.-H. Competing electronic orders on kagome lattices at van Hove filling. *Phys. Rev. B* **87**, 115135 (2013).

[14] Kiesel, M. L., Platt, C. & Thomale, R. Unconventional Fermi surface instabilities in the kagome Hubbard model. *Phys. Rev. Lett.* **110**, 126405 (2013). 25166827 10.1103/PhysRevLett.110.126405

[15] Yin, J.-X., Lian, B. & Hasan, M. Z. Topological kagome magnets and superconductors. *Nature* **612**, 647 (2022). 36543954 10.1038/s41586-022-05516-0

[16] Yin, J.-X. et al. Negative flat band magnetism in a spin–orbit-coupled correlated kagome magnet. *Nat. Phys.* **15**, 443 (2019).

[17] Yin, J.-X. et al. Spin-orbit quantum impurity in a topological magnet. *Nat. Commun.* **11**, 4415 (2020). 32887890 10.1038/s41467-020-18111-6 PMC7474094

[18] Huang, H. et al. Flat-band-induced anomalous anisotropic charge transport and orbital magnetism in kagome metal CoSn. *Phys. Rev. Lett.* **128**, 096601 (2022). 35302793 10.1103/PhysRevLett.128.096601

[19] Xing, Y. et al. Localized spin-orbit polaron in magnetic Weyl semimetal Co_3_Sn_2_S_2_. *Nat. Commun.* **11**, 5613 (2020). 33154384 10.1038/s41467-020-19440-2 PMC7644724

[20] Fenner, L., Dee, A. & Wills, A. Non-collinearity and spin frustration in the itinerant kagome ferromagnet Fe_3_Sn_2_. *J. Phys. Condens. Matter* **21**, 452202 (2009). 21694002 10.1088/0953-8984/21/45/452202

[21] Hou, Z. et al. Observation of various and spontaneous magnetic skyrmionic bubbles at room temperature in a frustrated kagome magnet with uniaxial magnetic anisotropy. *Adv. Mater.* **29**, 1701144 (2017). 10.1002/adma.201701144 28589629

[22] Yu, S.-L. & Li, J.-X. Chiral superconducting phase and chiral spin-density-wave phase in a Hubbard model on the kagome lattice. *Phys. Rev. B* **85**, 144402 (2012).

[23] Ye, L. et al. Hopping frustration-induced flat band and strange metallicity in a kagome metal. *Nat. Phys.* **20**, 610–614 (2024).

[24] Mozaffari, S. et al. Universal sublinear resistivity in vanadium kagome materials hosting charge density waves. *Phys. Rev. B* **110**, 035135 (2024).

[25] Ortiz, B. R. et al. CsV_3_Sb_5_: a topological kagome metal with a superconducting ground state. *Phys. Rev. Lett.* **125**, 247002 (2020). 33412053 10.1103/PhysRevLett.125.247002

[26] Ortiz, B. R. et al. Superconductivity in the kagome metal KV_3_Sb_5_. *Phys. Rev. Mater.* **5**, 034801 (2021).

[27] Wang, N. N. et al. Competition between charge-density-wave and superconductivity in the kagome metal RbV_3_Sb_5_. *Phys. Rev. Res.* **3**, 043018 (2021).

[28] Yin, Q. et al. Superconductivity and normal-state properties of kagome metal RbV_3_Sb_5_ single crystals. *Chin. Phys. Lett.* **38**, 037403 (2021).

[29] Ortiz, B. R. et al. New kagome prototype materials: discovery of KV_3_Sb_5_, RbV_3_Sb_5_, and CsV_3_Sb_5_. *Phys. Rev. Mater.* **3**, 094407 (2019).

[30] Yang, Y. C. et al. Unveiling the charge density wave mechanism in vanadium-based Bi-layered kagome metals. *NPG Asia Mater* **16**, 46 (2024).

[31] Weiland, A. et al. Refine intervention: characterizing disordered Yb_0.5_Co_3_Ge_3_. *Cryst. Growth Des.* **20**, 6715 (2020).

[32] Wang, Y. et al. Electronic properties and phase transition in the kagome metal Yb_0.5_Co_3_Ge_3_. *Chem. Mater.* **34**, 7337 (2022).

[33] Sinha, M. et al. Twisting of 2D kagome sheets in layered intermetallics. *ACS Cent. Sci.* **7**, 1381 (2021). 34471681 10.1021/acscentsci.1c00599 PMC8393211

[34] Abrikosov, A. A. *Fundamentals of the Theory of Metals* (Courier Dover, 2017).

[35] Hwang, E. & Das Sarma, S. Linear-in-T resistivity in dilute metals: a Fermi liquid perspective. *Phys. Rev. B* **99**, 085105 (2019).

[36] Liu, Z. et al. Orbital-selective Dirac fermions and extremely flat bands in frustrated kagome-lattice metal CoSn. *Nat. Commun.* **11**, 4002 (2020). 32778641 10.1038/s41467-020-17462-4 PMC7417585

[37] Kang, M. et al. Topological flat bands in frustrated kagome lattice CoSn. *Nat. Commun.* **11**, 4004 (2020). 32778669 10.1038/s41467-020-17465-1 PMC7417556

[38] Meier, W. R. et al. Flat bands in the CoSn-type compounds. *Phys. Rev. B* **102**, 075148 (2020).

[39] Ortiz, B. R. et al. Fermi surface mapping and the nature of charge-density-wave order in the kagome superconductor CsV_3_Sb_5_. *Phys. Rev. X* **11**, 041030 (2021).

[40] Tan, H. & Yan, B. Abundant lattice instability in kagome metal ScV_6_Sn_6_. *Phys. Rev. Lett.* **130**, 266402 (2023). 37450790 10.1103/PhysRevLett.130.266402

[41] Zheng, G. et al. Quantum oscillations evidence for topological bands in kagome metal ScV_6_Sn_6_. *J. Phys. Condens. Matter* **36**, 215501 (2024). 10.1088/1361-648X/ad2803 38335546

[42] Liu, Z. et al. Charge-density-wave-induced bands renormalization and energy gaps in a kagome superconductor RbV_3_Sb_5_. *Phys. Rev. X* **11**, 041010 (2021).

[43] González, J., Guinea, F. & Vozmediano, M. A. H. Unconventional quasiparticle lifetime in graphite. *Phys. Rev. Lett.* **77**, 3589 (1996). 10062258 10.1103/PhysRevLett.77.3589

[44] Hofmann, J., Barnes, E. & Das Sarma, S. Interacting Dirac liquid in three-dimensional semimetals. *Phys. Rev. B* **92**, 045104 (2015).

[45] Fritz, L., Schmalian, J., Müller, M. & Sachdev, S. Quantum critical transport in clean graphene. *Phys. Rev. B* **78**, 085416 (2008).

[46] Gan, Y. et al. Magneto-Seebeck effect and ambipolar Nernst effect in the csv_3_sb_5_ superconductor. *Phys. Rev. B* **104**, L180508 (2021).

[47] Kuo, C.-N. et al. Effects of lattice instability on the thermoelectric behavior of kagome metal ScV_6_Sn_6_. *Appl. Phys. Lett.* **125**, 152202 (2024).

[48] Isobe, H. & Fu, L. Supermetal. *Phys. Rev. Res.* **1**, 033206 (2019).

[49] Oriekhov, D., Gusynin, V. & Loktev, V. Orbital susceptibility of T-graphene: interplay of high-order van Hove singularities and Dirac cones. *Phys. Rev. B* **103**, 195104 (2021).

[50] Classen, L., Chubukov, A. V., Honerkamp, C. & Scherer, M. M. Competing orders at higher-order Van Hove points. *Phys. Rev. B* **102**, 125141 (2020).

[51] Guinea, F. Charge distribution and screening in layered graphene systems. *Phys. Rev. B* **75**, 235433 (2007).

[52] Kiselev, E. I. & Schmalian, J. Nonlocal hydrodynamic transport and collective excitations in Dirac fluids. *Phys. Rev. B* **102**, 245434 (2020).

[53] Liu, G.-Z. & Cheng, G. Chiral symmetry breaking and violation of the Wiedemann-Franz law in underdoped cuprates. *Phys. Rev. B* **66**, 100505 (2002).

[54] Dóra, B. Wiedemann-Franz law in the *S**U*(*N*) Wolff model. *Phys. Rev. B* **74**, 161101 (2006).

[55] Sharapov, S. G., Gusynin, V. P. & Beck, H. Transport properties in the d-density-wave state in an external magnetic field: the Wiedemann-Franz law. *Phys. Rev. B* **67**, 144509 (2003).

[56] Koepernik, K. & Eschrig, H. Full-potential nonorthogonal local-orbital minimum-basis band-structure scheme. *Phys. Rev. B* **59**, 1743 (1999).

[57] Perdew, J. P., Burke, K. & Ernzerhof, M. Generalized gradient approximation made simple. *Phys. Rev. Lett.* **77**, 3865 (1996). 10062328 10.1103/PhysRevLett.77.3865

[58] Kundu, A. K. et al. Low-energy electronic structure in the unconventional charge-ordered state of ScV_6_Sn_6_. *Nat. Commun.* **15**, 5008 (2024). 38866766 10.1038/s41467-024-48883-0 PMC11169263

[59] Shrestha, K. et al. Electronic properties of kagome metal ScV_6_Sn_6_ using high-field torque magnetometry. *Phys. Rev. B* **108**, 245119 (2023).

[60] Wenzel, M. et al. Optical study of RbV_3_Sb_5_: multiple density-wave gaps and phonon anomalies. *Phys. Rev. B* **105**, 245123 (2022).

[61] Hu, Y. et al. Rich nature of van Hove singularities in kagome superconductor CsV_3_Sb_5_. *Nat. Commun.* **13**, 2220 (2022). 35468883 10.1038/s41467-022-29828-x PMC9038924

[62] Kato, T. et al. Surface-termination-dependent electronic states in kagome superconductors AV_3_ Sb_5_ (A = K, Rb, Cs) studied by micro-ARPES. *Phys. Rev. B* **107**, 245143 (2023).

[63] Yu, F. H. et al. Concurrence of anomalous Hall effect and charge density wave in a superconducting topological kagome metal. *Phys. Rev. B* **104**, L041103 (2021).

[64] Kato, T. et al. Three-dimensional energy gap and origin of charge-density wave in kagome superconductor KV_3_Sb_5_. *Commun. Mater.* **3**, 30 (2022).

[65] Yang, S.-Y. et al. Giant, unconventional anomalous Hall effect in the metallic frustrated magnet candidate, KV_3_Sb_5_. *Sci. Adv.* **6**, eabb6003 (2020). 32789181 10.1126/sciadv.abb6003 PMC7399694

[66] Tan, H., Jiang, Y., McCandless, G. T., Chan, J. Y. & Yan, B. Three-dimensional higher-order saddle-point-induced flatbands in Co-based kagome metals. *Phys. Rev. Res.* **6**, 043132 (2024).

