# Supplementary material for: Sublinear transport in Kagome metals from the interplay of Dirac cones and Van Hove singularities
Source: Nat Commun. 2025 Dec 5;16:11056. doi: 10.1038/s41467-025-65685-0 (PMC12698808; doi:10.1038/s41467-025-65685-0)
Supplement: Supplementary file 1 — Supplementary Information [file 41467_2025_65685_MOESM1_ESM.pdf]

Supplementary Information for 'Sublinear transport in Kagome metals from the interplay of Dirac cones and Van Hove singularities'

Nikolai Peshcherenko,[1] Ning Mao,[1] Claudia Felser,[1] and Yang Zhang[2,3,*]
[1]Max Planck Institute for Chemical Physics of Solids, 01187, Dresden, Germany
[2]Department of Physics and Astronomy, University of Tennessee, Knoxville, Tennessee 37996, USA
[3]Min H. Kao Department of Electrical Engineering and Computer Science, University of Tennessee, Knoxville, Tennessee 37996, USA

I. SUBLINEAR IN TEMPERATURE TRANSPORT: ANALYTICAL DERIVATION

A. Internode scattering time evaluation

In this section, we provide a semiclassical derivation of electron-electron scattering rate and transport coefficients within the Boltzmann equation framework. Kinetic equations for distribution functions of fast Dirac electrons f_d and slow VHS electrons f_v are given by

$$\partial_t f_d + \mathbf{v}\cdot\partial_{\mathbf{r}} f_d + e\mathbf{E}\cdot\partial_{\mathbf{p}} f_d = I^d_{\text{imp}}\left[f_d\right] + I^d_{\text{e}}\left[f_d,\, f_v\right], \tag{1}$$

$$\partial_t f_v + \mathbf{v}\cdot\partial_{\mathbf{r}} f_v + e\mathbf{E}\cdot\partial_{\mathbf{p}} f_v = I^v_{\text{imp}}\left[f_v\right] + I^v_{\text{e}}\left[f_d,\, f_v\right]. \tag{2}$$

Here I_{imp} describes impurity scattering, I_{e} - electron-electron scattering. The crucial ingredient of our theory lies in the electron-electron scattering part of collision integral I_{e}:

$$I^d_{\text{e}}\left[f_d,\, f_v\right] = I^d_{\text{e}}\left[f_d,\, f_v\right]_{\text{in}} - I^d_{\text{e}}\left[f_d,\, f_v\right]_{\text{out}},$$
$$I^d_{\text{e}}\left[f_d,\, f_v\right]_{\text{in}} = \sum_{\mathbf{p}'} W_{\mathbf{p}'\to\mathbf{p}}\, f_d(\mathbf{p}')\left(1-f_d(\mathbf{p})\right), \quad I^d_{\text{e}}\left[f_d,\, f_v\right]_{\text{out}} = \sum_{\mathbf{p}'} W_{\mathbf{p}\to\mathbf{p}'}\cdot f_d(\mathbf{p})\left(1-f_d(\mathbf{p}')\right). \tag{3}$$

In turn, transition probabilities $W_{\mathbf{p}\to\mathbf{p}'}$, $W_{\mathbf{p}'\to\mathbf{p}}$ are given by

$$W_{\mathbf{p}'\to\mathbf{p}} = 2\pi g^2 \sum_{\mathbf{p_1},\mathbf{p_2}} f_v(\mathbf{p_1})\left(1-f_v(\mathbf{p_2})\right)\delta\left(E_d(\mathbf{p}) + E_v(\mathbf{p_2}) - E_d(\mathbf{p}') - E_v(\mathbf{p_1})\right)\delta\left(\mathbf{p}+\mathbf{p}_2-\mathbf{p}'-\mathbf{p}_1\right),$$
$$W_{\mathbf{p}\to\mathbf{p}'} = 2\pi g^2 \sum_{\mathbf{p_1},\mathbf{p_2}} f_v(\mathbf{p_2})\left(1-f_v(\mathbf{p_1})\right)\delta\left(E_d(\mathbf{p}') + E_v(\mathbf{p_1}) - E_d(\mathbf{p}) - E_v(\mathbf{p_2})\right)\delta\left(\mathbf{p}'+\mathbf{p}_1-\mathbf{p}-\mathbf{p}_2\right). \tag{4}$$

In Eqs. (3), (4) $\mathbf{p},\mathbf{p}'$ are initial and final momenta of a Dirac electron, $\mathbf{p_1},\mathbf{p_2}$ are the same but for VHS electron. The equation (4) itself is very general and can describe, for instance, the standard T^2 metal behavior either for intra- or interpocket scattering processes (including Umklapp processes). However, in Kagome metal case $E_v(\mathbf{p})$ demonstrates a very weak dispersion, meaning that one can adopt $E_v(\mathbf{p}_1) \approx E_v(\mathbf{p}_2)$.

In order to make it easier to compare with calculations of previous sections change momentum variables from $\mathbf{p}_1$, $\mathbf{p}_2$, $\mathbf{p}'$ to the following ones:

$$\mathbf{p}' = \mathbf{p}+\mathbf{q}, \quad \mathbf{p}_2 = \mathbf{p}_v - \mathbf{q}, \quad \mathbf{p}_v = \mathbf{p}_1. \tag{5}$$

In these notations, momentum conservation law is fullfiled automatically. For transition probabilities from (4) we then arrive at

$$W_{\mathbf{p}'\to\mathbf{p}} \approx 2\pi g^2 \sum_{\mathbf{p}_v} f_v(\mathbf{p}_v)\left(1-f_v(\mathbf{p}_v+\mathbf{q})\right)\delta\left(E_d(\mathbf{p}) - E_d(\mathbf{p}+\mathbf{q})\right),$$
$$W_{\mathbf{p}\to\mathbf{p}'} \approx 2\pi g^2 \sum_{\mathbf{p}_v} f_v(\mathbf{p}_v)\left(1-f_v(\mathbf{p}_v-\mathbf{q})\right)\delta\left(E_d(\mathbf{p}) - E_d(\mathbf{p}+\mathbf{q})\right) \tag{6}$$

* Corresponding author, e-mail yangzhang@utk.edu

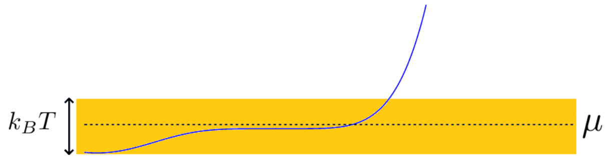

FIG. 1. A sketch of Van Hove singularity in Ni_3In. Yellow: thermally activated electrons. For high temperatures ($T \gtrsim 100\,\mathrm{K}$) number of activated electrons does not depend on T.

Since for equilibrium distribution function $f_v(\mathbf{k}) = f_v(-\mathbf{k})$, we find in accord with detailed balance principle

$$W_{\mathbf{p}'\to\mathbf{p}} = W_{\mathbf{p}\to\mathbf{p}'} = 2\pi g^2 F(\mathbf{q},T)\delta\left(E_d(\mathbf{p}) - E_d(\mathbf{p}+\mathbf{q})\right),$$

$$F(\mathbf{q},T) \equiv \sum_{\mathbf{p}_v} f_v(\mathbf{p}_v)\left(1 - f_v(\mathbf{p}_v - \mathbf{q})\right) = \int \frac{d^2\mathbf{p}_v}{(2\pi)^2}\frac{1}{\left(e^{\beta(E_v(\mathbf{p}_v)-\mu_v)}+1\right)\left(e^{-\beta(E_v(\mathbf{p}_v-\mathbf{q})-\mu_v)}+1\right)} \tag{7}$$

so that

$$I_e^d\left[f_d\right] = \sum_{\mathbf{q}} W_{\mathbf{p}+\mathbf{q}\to\mathbf{p}}\cdot f_d(\mathbf{p}+\mathbf{q})\left(1 - f_d(\mathbf{p})\right) - \sum_{\mathbf{q}} W_{\mathbf{p}\to\mathbf{p}+\mathbf{q}}\cdot f_d(\mathbf{p})\left(1 - f_d(\mathbf{p}+\mathbf{q})\right) =$$
$$= 2\pi g^2 \sum_{\mathbf{q}} F(\mathbf{q},T)\delta\left(E_d(\mathbf{p}) - E_d(\mathbf{p}+\mathbf{q})\right)\left(f_d(\mathbf{p}+\mathbf{q}) - f_d(\mathbf{p})\right). \tag{8}$$

Substituting $f_d(\mathbf{p}) = f_{\mathrm{eq}}(|\mathbf{p}|) + \delta f(\mathbf{p})$ into Eq. (3) and evaluating the integral in polar coordinates, we arrive at:

$$I_e^d\left[f_d\right] = g^2\int_0^{2\pi}\frac{d\varphi}{2\pi}\int q dq\,\delta\left[v_F\left(p_F - \sqrt{p_F^2 + q^2 + 2p_F q\cos\varphi}\right)\right]F(\mathbf{q},T)\left(\delta f(\mathbf{p}+\mathbf{q}) - \delta f(\mathbf{p})\right) =$$
$$= \frac{g^2}{2\pi v_F}\int dq\frac{\theta(2p_F - q)}{\sqrt{1 - q^2/4p_F^2}}F(q,\varphi_q,T)\left(\delta f(\mathbf{p}+\mathbf{q}) - \delta f(\mathbf{p})\right)|_{\varphi=\varphi_q}. \tag{9}$$

In order to arrive at a reasonable analytical estimate of Eq. (9), it is required to perform an estimate of $F(\mathbf{q})$ defined by Eq. (7). Let us first of all note that outside of the region $q \le q_T \equiv (T/A)^{1/\alpha}$ function $F(\mathbf{q})$ should decay exponentially. Now, having in mind Ni_3In case (see Fig. 4a of the main text), it is easy to see that for $q \le q_T$ and $T \gg \mu_v$ VHS electrons should obey Boltzmann statistics (see also Fig.1). Hence,

$$F(\mathbf{q},T) = \sum_{\mathbf{p}_v} f_v(\mathbf{p}_v)\left(1 - f_v(\mathbf{p}_v - \mathbf{q})\right) \approx \theta\left(q_T - |\mathbf{q}|\right)\sum_{\mathbf{p}_v} f_v(\mathbf{p}_v) = n_v\theta\left(q_T - |\mathbf{q}|\right), \tag{10}$$

where n_v is the total VHS electrons concentration. Therefore, one can say that for Ni_3In material case $F(\mathbf{q},T)$ is actually T-independent and gives simply n_v.

Although having an ideal saddle point-type of Van Hove singularity (i.e., uncut from below, see band structures of section II) would most probably result in having a T-dependent n_v, the details of this dependence would be determined by electron bands behavior for energies below the Fermi surface (or, alternatively, on the line between M and K points in momentum space). Given that the upper bands at Fig. 3 demonstrate weak momentum dependence for the states below Fermi energy, one could also expect weak T-dependence of n_v for other Kagome materials.

Now, making use of the Eq. (10) result and assuming small thermally activated Fermi surface $q_T \ll k_F$ (this could be proven to be experimentally relevant for electrons concentration values from [1] and real material band structures of section II) and a standard Ansatz for $\delta f(\mathbf{p}) = \frac{\mathbf{E}\mathbf{p}}{|\mathbf{p}|}\chi(|\mathbf{p}|)$, we arrive at

$$I_e^d\left[f_d\right] = -\frac{\chi}{\tau_{\mathrm{e-e}}},\quad \frac{1}{\tau_{\mathrm{e-e}}} \sim \frac{g^2 n_v}{\mu_d^2/v_F}\left(\frac{T}{A}\right)^{3/\alpha}. \tag{11}$$

B. Long range Coulomb scattering

As a side note, we would also like to showcase that even in the presence of long-ranged Coulomb interaction $V(\mathbf{q}) = \frac{2\pi e^2}{|\mathbf{q}|}$ the scattering time remains sublinear. The general expression for transport scattering time τ_{tr} reads

$$\frac{1}{\tau_{\mathrm{tr}}} \propto \int_{|\mathbf{q}|<q_T} d^2\mathbf{q}|V(\mathbf{q})|^2\delta\left(E(\mathbf{p})-E(\mathbf{p}+\mathbf{q})\right)(1-\cos\theta_{\mathbf{p},\mathbf{p}+\mathbf{q}}) \propto$$

$$\propto \int_0^{q_T} \frac{dq}{q^2\sqrt{1-q^2/4p_F^2}}\left(\frac{q}{2p_F}\right)^2 \propto q_T \propto \left(\frac{T}{A}\right)^{1/\alpha}, \tag{12}$$

still demonstrating the sublinear scaling with temperature T.

C. Transport coefficients derivation

Given the collision integral from the Eq. (11), one can follow a well-known textbook procedure [2] for finding kinetic coefficients. Linear in $\mathbf{E}$, ∇T approximation of Boltzmann could then be written as

$$\mathbf{v}\cdot\left[e\mathbf{E}-\frac{\varepsilon-\mu_d}{T}\nabla T\right]\partial_\varepsilon f_{\mathrm{eq}} = -\frac{\delta f_d}{\tau_{\mathrm{imp}}}-\frac{\delta f_d}{\tau_{\mathrm{inter}}} \tag{13}$$

so that its solution reads

$$\delta f_d = e\tau_{\mathrm{eff}}\left(-\partial_\varepsilon f_{\mathrm{eq}}\right)\mathbf{v}\cdot\left[e\mathbf{E}-\frac{\varepsilon-\mu_d}{T}\nabla T\right], \quad \tau_{\mathrm{eff}} = \left(\tau_{\mathrm{imp}}^{-1}+\tau_{\mathrm{inter}}^{-1}\right)^{-1} \tag{14}$$

and for charge and heat currents $\mathbf{j}$, $\mathbf{j_q}$ of Dirac electrons we arrive at

$$\mathbf{j} = e^2\sum_{\mathbf{p}}\mathbf{v}_d\left(\mathbf{v}_d\mathbf{E}\right)\tau_{\mathrm{eff}}\left(-\partial_\varepsilon f_{\mathrm{eq}}\right), \quad \mathbf{j_q} = -\sum_{\mathbf{p}}\frac{(\varepsilon_d-\mu_d)^2}{T}\mathbf{v}_d\left(\mathbf{v}_d\nabla T\right)\tau_{\mathrm{eff}}\left(-\partial_\varepsilon f_{\mathrm{eq}}\right), \tag{15}$$

which for $T \ll \mu_d$ gives standard semiclassical results

$$\sigma = e^2\nu_d(\mu_d)D, \quad \kappa = \frac{\pi^2}{3}\nu_d(\mu_d)DT, \quad D = \frac{1}{2}v_F^2\tau_{\mathrm{eff}}(\mu_d, T). \tag{16}$$

II. REAL MATERIAL DATA COMPARISON: $\mathrm{SCV_6SN_6}$, $\mathrm{CSV_3SB_5}$, $\mathrm{RBV_3SB_5}$ AND $\mathrm{KV_3SB_5}$

In addition to the Ni_3In experiments mentioned in the main text, in this appendix we give consideration to other Kagome metals that demonstrated [1, 3–7] similar sublinear scaling of resistivity with temperature $\rho(T) \propto T^\gamma$, $\gamma = 0.62$. From FPLO-calculated band structure for ScV_6Sn_6, CsV_3Sb_5, RbV_3Sb_5, KV_3Sb_5 (see Fig. 2) one can see that all of these materials have a fast Dirac pocket near K point and a saddle point type of Van Hove singularity around M point, thus making a ground for a simple two-pocket approach developed in the main text.

Our prediction for resistivity scaling exponent (see Eq. (8) of the main text) heavily depends on the bands behavior near M point. Thus, in order to compare it with experimental results [1] we performed $k\cdot p$ model fitting for the two DFT bands closest to the Fermi level near M point (see Fig. 3).

We fit the bands near the Fermi level using two polynomial functions, each with a maximum order of five. These polynomials are represented as:

$$H(\mathbf{k}) = \begin{pmatrix} P_1(\mathbf{k}) & 0 \\ 0 & P_2(\mathbf{k}) \end{pmatrix}, \tag{17}$$

where $P_1(\mathbf{k})$, $P_2(\mathbf{k})$ are given by

$$\begin{aligned} P_1(\mathbf{k}) &= a_1k_x^5 + a_2k_x^3 + a_3k_xk_y^2 + a_4k_yk_x^2 + a_5 \\ P_2(\mathbf{k}) &= b_1k_x^5 + b_2k_x^3 + b_3k_x^2k_y + b_4k_xk_y^2 + b_5. \end{aligned} \tag{18}$$

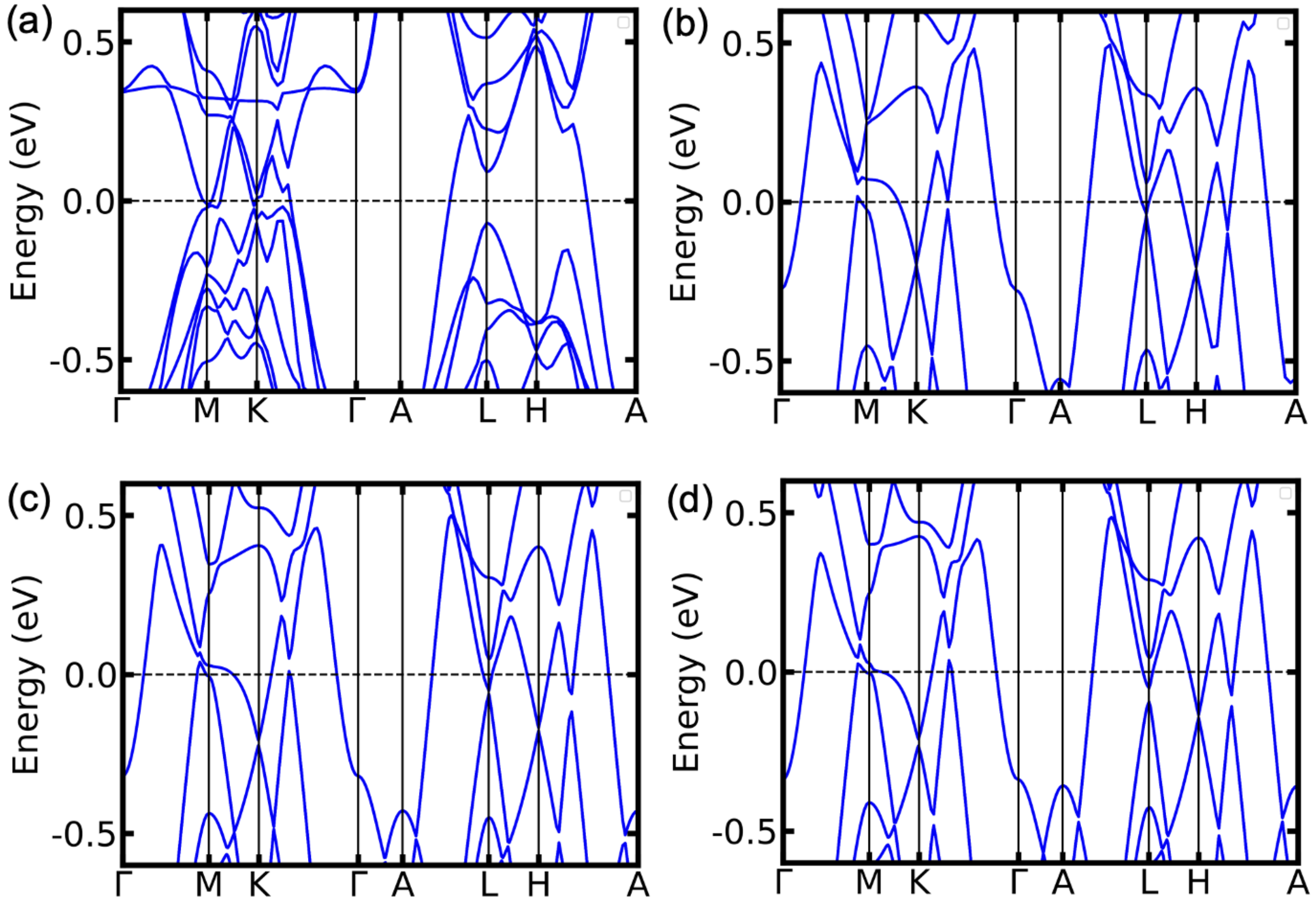

FIG. 2. The band structure of (a) ScV_6Sn_6, (b) CsV_3Sb_5, (c) RbV_3Sb_5 and (d) KV_3Sb_5. All materials listed demonstrate having a fast Dirac pocket near K point and a saddle point type of Van Hove singularity around M point.

Please note that although these compounds have P6/mmm symmetry group, for a two-band modelling not all of the group symmetries could be respected. Thus, we keep inversion symmetry that allows only for odd powers of k. By fitting the band structures from FPLO calculations with Eq. (18) we can obtain the 10 expansion coefficients (please see Table I).

Material	a_1	a_2	a_3	a_4	a_5	b_1	b_2	b_3	b_4	b_5
ScV_6Sn_6	0.00319	-0.0533	-0.0789	-0.000259	0.710	-0.00248	0.0402	0.00176	0.168	-0.484
CsV_3Sb_5	0.00170	-0.0283	-0.0274	-0.0000193	0.425	0.00434	-0.0726	-0.00580	-0.265	0.897
RbV_3Sb_5	0.00139	-0.0261	-0.247	-0.00788	0.311	0.00493	-0.0787	0.000792	-0.0497	0.893
KV_3Sb_5	0.00167	-0.0281	-0.281	0.00472	0.354	0.00446	-0.0736	-0.0136	-0.0135	0.947

TABLE I. Result of a corresponding material DFT bands fitting with Eq. (18).

In 3D material case power-law type of VHS (see calculated density of states at Fig. 3) could be provided only by 5th order term. Thus, our DFT bands fitting, according to Eq. (8) of the main text, predicts 0.6 resistivity exponent which stands in full agreement with experimental value $n = 0.62$ from [1].

III. ANOMALOUS HALL EFFECT

Yet another physical aspect a high order Van Hove singularity could bring to transport features is anomalous Hall response. This phenomenon was recently observed experimentally in various Kagome materials [8–11], where it was

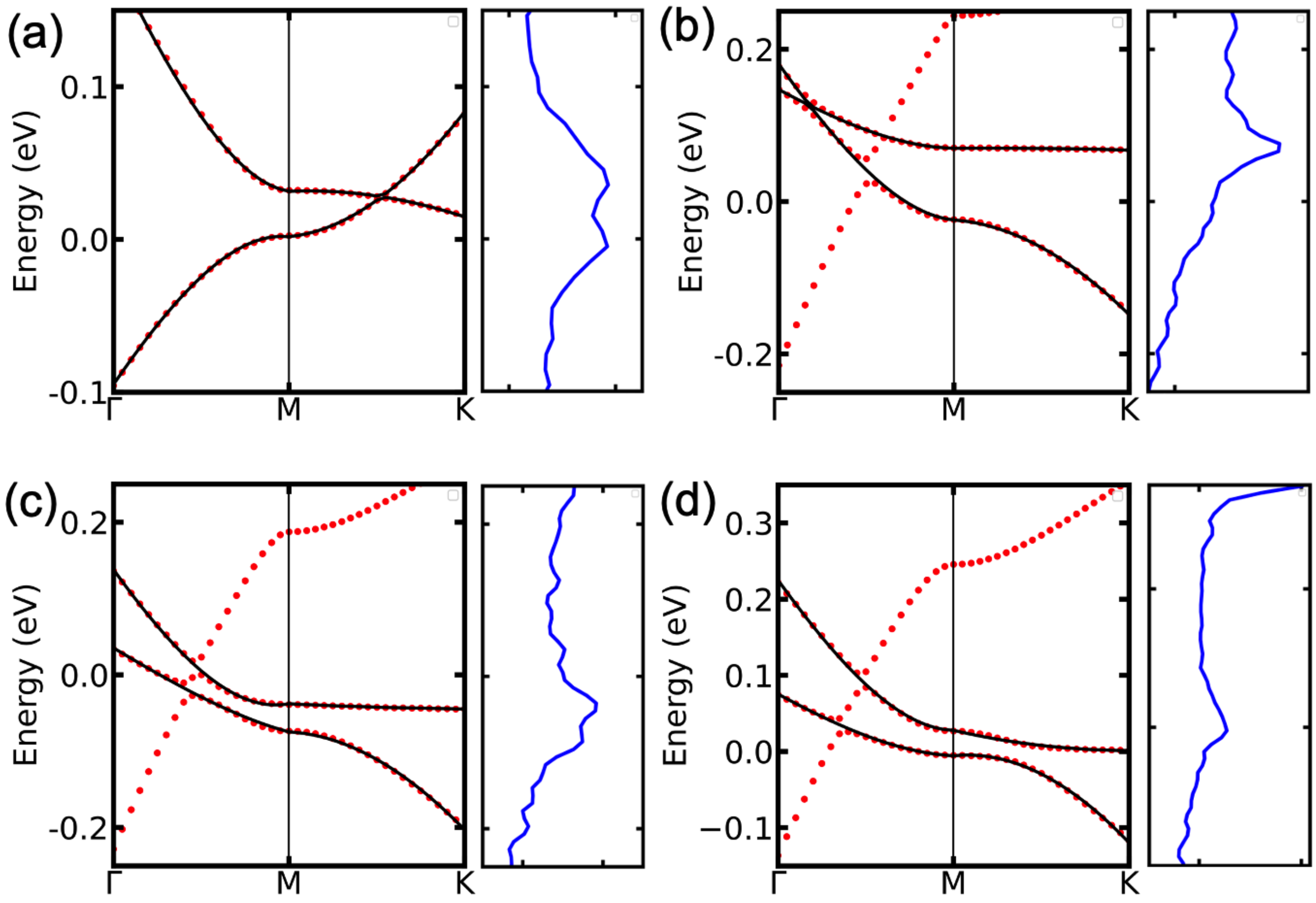

FIG. 3. The fitted band Structure of (a) ScV_6Sn_6, (b) CsV_3Sb_5, (c) RbV_3Sb_5, and (d) KV_3Sb_5. The black lines represent the $k \cdot p$ model's prediction, while the red dots indicate the FPLO-calculated band structures in the vicinity of the M points. Notably, there is a switch in the representations around the Dirac point. Calculated density of states is shown by blue line. One can see that in all cases it has a peak near Fermi level.

attributed either to intrinsic (Berry curvature-related) or extrinsic (skew scattering) contributions. However, first-principle calculations conducted for KV_3Sb_5 [12] predicts that an intrinsic contribution is much smaller than the one observed in experiment [9]. And it has been shown [13] that KV_3Sb_5 does not support any net magnetic moment so that there is no ground for skew scattering. Hence, other mechanisms should be considered.

We suggest that the role of magnetic impurities could be played by Van Hove singularity electrons. Namely, we imply that Stoner instability for VHS pocket electrons may occur due to diverging density of states. Thus, scattering of fast Dirac electrons at VHS states would allow for a skew-scattering contribution off the electrons magnetic moment.

One can estimate the effective electron exchange constant U to be of the order of $U \sim \nu_v^{-1}$, hence $\tau_{\rm skew}$ is given by

$$\frac{1}{\tau_{\rm skew}} \sim \frac{1}{\tau_{\rm tr}} U \nu_d(\mu) \sim \frac{1}{\tau_{\rm tr}} \frac{\nu_d(\mu)}{\nu_v(\mu)}, \tag{19}$$

so that

$$\frac{\rho_H}{\rho_0} \sim \frac{\tau_{\rm tr}}{\tau_{\rm skew}} \tag{20}$$

where ρ_H and ρ_0 are anomalous Hall and longitudinal resistivity correspondingly. Employing the result of Eq. (8), for T-dependent Hall conductivity $\rho_H(T)$ (assuming that $\mu_v \ll T$) we arrive at

$$\rho_H(T) \propto \begin{cases} T^{1+1/\alpha}, & \mu_d \gg T \\ T^{1+d+1/\alpha}, & \mu_d \ll T, \end{cases} \tag{21}$$

where $d = 2, 3$ is crystal dimension. One can see that deviations from T-linear behavior for anomalous Hall resistivity ρ_{xy} could be considered as a probe of electronic band structure.

IV. SCHEMATIC LORENTZ NUMBER T BEHAVIOR

The behavior of $L(T)$ is presented at Fig. 5 of the main text. Note also that even though Kagome metals are bulk materials, their band structure is still highly anisotropic and quasi-2D like. Therefore, for Fig. 5 we decided to keep both $d = 2$ and $d = 3$ cases, so that real material behavior should lay in between. $T_{\rm cr}$ is a crossover temperature between impurity scattering-mediated and internode scattering-mediated τ_κ, where we assumed that impurity scattering happens at short-ranged impurities, so that $\tau^{-1}_{\rm imp}(T) \propto \nu_d(T)$. For $d = 2$ the Eq. (10) of the main text predicts saturation of $L(T)$ for $T > T_{\rm cr}$. However, this saturation behavior would switch to a decrease if one considers electron-phonon coupling at higher T close to Debye temperature.

---

[1] S. Mozaffari, W. R. Meier, R. P. Madhogaria, N. Peshcherenko, S.-H. Kang, J. W. Villanova, H. W. S. Arachchige, G. Zheng, Y. Zhu, K.-W. Chen, *et al.*, Universal sublinear resistivity in vanadium kagome materials hosting charge density waves, Physical Review B **110**, 035135 (2024).

[2] L. P. Pitaevskii and E. Lifshitz, *Physical Kinetics: Volume 10*, Butterworth-Heinemann, 2012.

[3] B. R. Ortiz, S. M. L. Teicher, Y. Hu, J. L. Zuo, P. M. Sarte, E. C. Schueller, A. M. M. Abeykoon, M. J. Krogstad, S. Rosenkranz, R. Osborn, R. Seshadri, L. Balents, J. He, and S. D. Wilson, CsV_3Sb_5: A $\mathbb{Z}_2$ Topological Kagome Metal with a Superconducting Ground State, Physical Review Letters **125**, 247002 (2020).

[4] B. R. Ortiz, P. M. Sarte, E. M. Kenney, M. J. Graf, S. M. L. Teicher, R. Seshadri, and S. D. Wilson, Superconductivity in the $\mathbb{Z}_2$ kagome metal KV_3Sb_5, Physical Review Materials **5**, 034801 (2021).

[5] N. N. Wang, K. Y. Chen, Q. W. Yin, Y. N. N. Ma, B. Y. Pan, X. Yang, X. Y. Ji, S. L. Wu, P. F. Shan, S. X. Xu, Z. J. Tu, C. S. Gong, G. T. Liu, G. Li, Y. Uwatoko, X. L. Dong, H. C. Lei, J. P. Sun, and J.-G. Cheng, Competition between charge-density-wave and superconductivity in the kagome metal RbV_3Sb_5, Physical Review Research **3**, 043018 (2021).

[6] Q. Yin, Z. Tu, C. Gong, Y. Fu, S. Yan, and H. Lei, Superconductivity and normal-state properties of kagome metal RbV_3Sb_5 single crystals, Chinese Physics Letters **38**, 037403 (2021).

[7] B. R. Ortiz, L. C. Gomes, J. R. Morey, M. Winiarski, M. Bordelon, J. S. Mangum, I. W. Oswald, J. A. Rodriguez-Rivera, J. R. Neilson, S. D. Wilson, *et al.*, New kagome prototype materials: discovery of KV_3Sb_5, RbV_3Sb_5, and CsV_3Sb_5, Physical Review Materials **3**, 094407 (2019).

[8] C. Yi, X. Feng, N. Mao, P. Yanda, S. Roychowdhury, Y. Zhang, C. Felser, and C. Shekhar, Quantum oscillations revealing topological band in kagome metal ScV_6Sn_6, Physical Review B **109**, 035124 (2024).

[9] F. H. Yu, T. Wu, Z. Y. Wang, B. Lei, W. Z. Zhuo, J. J. Ying, and X. H. Chen, Concurrence of anomalous Hall effect and charge density wave in a superconducting topological kagome metal, Phys. Rev. B **104**, L041103 (2021).

[10] S.-Y. Yang, Y. Wang, B. R. Ortiz, D. Liu, J. Gayles, E. Derunova, R. Gonzalez-Hernandez, L. Smejkal, Y. Chen, S. S. Parkin *et al.*, Giant, unconventional anomalous Hall effect in the metallic frustrated magnet candidate, KV_3Sb_5, Science advances **6**, eabb6003 (2020).

[11] Y. Wang, Z. Chen, Y. Nie, Y. Zhang, Q. Niu, G. Zheng, X. Zhu, W. Ning, and M. Tian, Nontrivial Fermi surface topology and large anomalous Hall effect in the kagome superconductor RbV_3Sb_5, Phys. Rev. B **108**, 035117 (2023).

[12] Y.-X. Jiang, J.-X. Yin, M. M. Denner, N. Shumiya, B. R. Ortiz, G. Xu, Z. Guguchia, J. He, M. S. Hossain, X. Liu, *et al.*, Unconventional chiral charge order in kagome superconductor KV_3Sb_5, Nature materials **20**, 1353 (2021).

[13] E. M. Kenney, B. R. Ortiz, C. Wang, S. D. Wilson, and M. J. Graf, Absence of local moments in the kagome metal KV_3Sb_5 as determined by muon spin spectroscopy, Journal of Physics: Condensed Matter **33**, 235801 (2021).
